# Role of inflammasomes in *Toxoplasma* and *Plasmodium* infections

**DOI:** 10.1186/s13071-024-06529-6

**Published:** 2024-11-15

**Authors:** Zhi-xin Wang, Wan-jun Jiao, Yong Yang, Hong-li Liu, Hai-long Wang

**Affiliations:** School of Basic Medicine, Basic Medical Sciences Center, Shanxi Medical University, Jinzhong, 030600 Shanxi China

**Keywords:** *T. gondii*, *Plasmodium*, Inflammasomes, Infection

## Abstract

**Background:**

The detection of pathogen-associated molecular patterns (PAMPs) or damage-associated molecular patterns (DAMPs) by multimeric protein complexes, known as inflammasomes, triggers an inflammatory response, which is a critical component of the innate immune system. This inflammatory response plays a pivotal role in host resistance against parasitic infections, presenting a significant global health challenge.

**Methods:**

We systematically searched for relevant articles from the Pubmed and the Web of Science database to summarize current insights into how inflammasomes function in preventing infections caused by the apicomplexan parasites *Toxoplasma* and *Plasmodium*.

**Results:**

In vivo and in vitro studies have extensively explored inflammasomes such as the absent in melanoma 2 (AIM2), NLR family pyrin-containing protein 1 (NLRP1), NLRP3, and NLRP12 inflammasomes, alongside noncanonical inflammasomes, with particular emphasis on the NLRP1 and the NLRP3 inflammasome during *Toxoplasma gondii * infection or the AIM2 and the NLRP3 inflammasome at various stages of *Plasmodium* infection. *Toxoplasma gondii* interacts with inflammasomes to activate or inhibit immune responses.

**Conclusions:**

Inflammasomes control parasite burden and parasite-induced cell death, contribute to immune recognition and inflammatory responses and thus influence apicomplexan parasite-associated pathogenesis and the severity of clinical outcomes. Hence, inflammasomes play crucial roles in the progression and outcomes of toxoplasmosis and malaria. A comprehensive understanding of how parasitic infections modulate inflammasome activity enhances insight into host immune responses against parasites.

**Graphical Abstract:**

**Figure:**
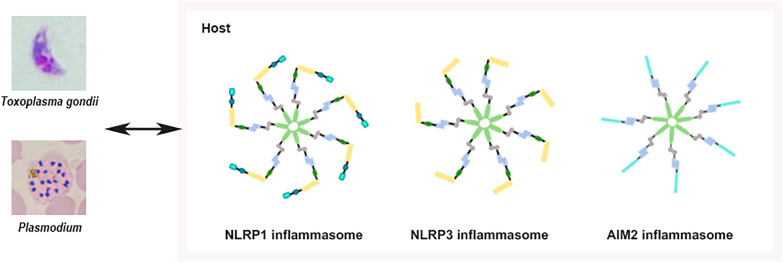

## Background

Inflammasomes, which are integral to the innate immune system, are indispensable for defending the host against pathogens and regulating the autoinflammatory response. Excessive inflammation can result in cell and tissue damage, whereas an insufficient inflammatory response may enable pathogens to persist in the host.

Inflammasomes represent a diverse group of cytoplasmic multiprotein complexes that primarily consist of a sensor protein, an adaptor protein and an inflammatory caspase. These sensor proteins, which function as pattern recognition receptors (PRRs), identify pathogen-associated molecular patterns (PAMPs) or damage-associated molecular patterns (DAMPs), initiating the assembly of inflammasomes. Common sensor proteins include Nucleotide binding oligomerization domain-like receptor (NLR) family proteins (such as NLRP1, NLRP3 and NLRC4), PYRIN and AIM2. Canonical inflammasomes (NLRP1, NLRP3, NLRC4, AIM2) activate caspase-1, leading to the release of the proinflammatory cytokines interleukin-1 beta (IL-1β) and IL-18 and the cleavage of gasdermin D (GSDMD) into its N-terminus (GSDMD-NT), triggering inflammation or cell death. The noncanonical inflammasomes caspase-11 (in mice) and caspase-4/5 (in humans) directly bind lipopolysaccharide (LPS) from Gram-negative bacteria, triggering caspase oligomerization and activation, which is independent of caspase-1 activation. Caspase-4/5/11 cannot directly process pro-IL-1β and pro-IL-18 into its mature fragment, but it can induce the secretion of IL-1β and IL-18 via NLRP3 inflammasome activation or cellular pyroptosis. NLR family proteins typically feature an leucine-rich repeat (LRR) domain that binds ligands, a central nucleotide-binding and oligomerization domain (NACHT) and an N-terminal caspase-activating and recruitment domain (CARD) or pyrin domain (PYD). Procaspase-1 is recruited to inflammasomes through its CARD domain by apoptosis-associated speck-like protein containing a CARD (ASC). The activation of the NLRP3 inflammasome involves two main phases: priming and activation. Priming involves PAMPs or DAMPs such as LPS, which activate nuclear factor kappa-light-chain-enhancer of activated B cells (NF-κB) signaling and induce the transcription of the NLRP3, IL-18 and IL-1β genes, accompanied by posttranslational modifications (ubiquitination, deubiquitination, phosphorylation and dephosphorylation of NLRP3). Activation signals (e.g. ATP, nigericin, free fatty acids, urate crystals and cholesterol crystals) trigger cellular responses, including potassium efflux, calcium mobilization, mitochondrial dysfunction, reactive oxygen species (ROS) production and lysosomal rupture.

NLRP3 inflammasome components are expressed across a spectrum of cell types, including monocytes, neutrophils, macrophages, dendritic cells, oligodendrocytes, astrocytes, endothelial cells and retinal pigmented epithelial cells. They are closely associated with the pathogenesis of various diseases, such as viral, fungal and bacterial infections; atherosclerosis; Alzheimer's disease; cancer; and nonalcoholic fatty liver disease [1]. These findings suggest that the NLRP3 inflammasome is an increasingly important target for treating inflammatory conditions. Recent studies highlight the crucial role of inflammasomes in the process of antiparasitic infection, as they participate in the inflammatory response against antiprotozoal infections and potentially aid in parasite clearance and reducing parasite loads. In toxoplasmosis and malaria, inflammasomes are primed and activated, responding to parasite components that act as PAMPs recognized by host PRRs, thereby regulating inflammasome activation. 

This review briefly summarizes recent developments in harnessing inflammasomes against *Toxoplasma* and *Plasmodium* infections.

## *Toxoplasma*

The obligate intracellular protozoan parasite *Toxoplasma gondii* infects virtually all nucleated cell types in warm-blooded animals, including humans, leading to toxoplasmosis. This zoonotic disease typically spreads through the ingestion of oocysts or tissue cysts (bradyzoites) in contaminated food or water. Global estimates indicate that approximately 30–50% of the world's population is infected with *T. gondii*, with seroprevalence rates varying widely (from < 10% to > 70%) across different regions [2, 3]. Despite their high prevalence, the majority of infected individuals either show no clinical symptoms or experience only mild discomfort. In these cases, the immune system controls the replication of *T. gondii* tachyzoites, while some tachyzoites may transform into slow-growing and metabolically inactive bradyzoites, primarily found in organs such as muscle and brain tissues, establishing chronic infections. However, in individuals with a suppressed or compromised immune system, bradyzoites can convert back into active tachyzoites, posing significant health risks [4]. Moreover, *T. gondii* infection in pregnant women can lead to miscarriage or congenital toxoplasmosis in newborns, impacting public health [5].

*Toxoplasma gondii* infection hinges on a delicate balance between the host immune response and parasite immune evasion strategies, enabling survival for both parties. Monocytes and macrophages, pivotal components of the innate immune system, are swiftly recruited to sites of *T. gondii* infection and play crucial roles in defending against *Toxoplasma.* Monocytes contribute by regulating parasite levels through the secretion of inflammatory cytokines, phagocytosis and antimicrobial effector mechanisms [6]. Moreover, macrophages curb the growth and reproduction of *T. gondii*, although the parasite can evade phagocytosis and invade macrophages by preventing the recruitment of immunity-related GTPases to the parasitophorous vacuole membrane [7].

**Fig. 1:**
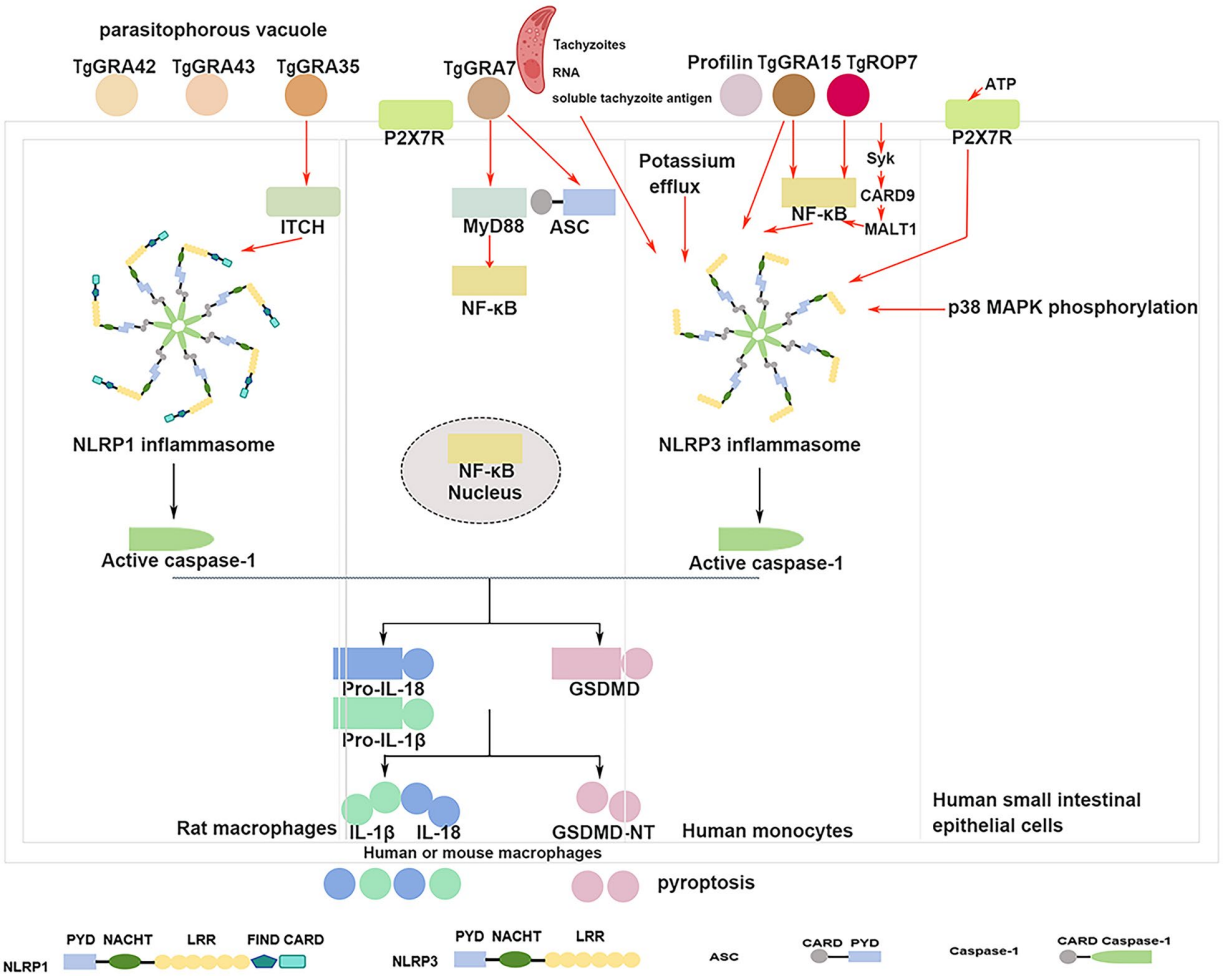
Inflammasomes mediate the immune response in host cells infected with *Toxoplasma gondii. Toxoplasma gondii* is involved primarily in NLRP1 and NLRP3 inflammasome activation in human macrophages, monocytes, small intestinal epithelial cells and mouse macrophages. *Tg*GRA7, *Tg*GRA15, profilin and *Tg*ROP7 are associated with NLRP1 and NLRP3 inflammasome activation. The P2X7 receptor and potassium efflux reportedly activate the NLRP3 inflammasome. *Toxoplasma gondii* activates a Syk-CARD9-NF-κB signaling axis, leading to NLRP3 inflammasome activation in primary human monocytes [9]. *Toxoplasma gondii*-induced NLRP3 inflammasome activation in human small intestinal epithelial cells is strongly correlated with the phosphorylation of p38 MAPK [10]. In addition, the NLRP1 inflammasome is activated in *T. gondii*-infected rat macrophages. *Tg*GRA35, *Tg*GRA42 and *Tg*GRA43 are associated with NLRP1 inflammasome activation [11]. CARD, Caspase-activating and recruitment domain; NF-κB, nuclear factor kappa-light-chain-enhancer of activated B cells; NLRP, NLR family pyrin-containing protein *Tg*GRA; *T. gondii* dense granule protein; *Tg*ROP7, *T. gondii* Rhoptry protein 7; see also Abbreviation list

Recent studies have revealed that *T. gondii*, an intracellular pathogen, has the ability to regulate inflammasome activation in a bidirectional manner. Upon invading immune cells, *T. gondii* can either induce inflammasome activation or inhibit inflammasome activation as part of its immune evasion strategy. The parasite manipulates human neutrophils to evade innate immunity, where infected neutrophils suppress LPS-induced IL-1β and NLRP3 transcription, reduce the expression of pro-IL-1β and mature IL-1β and hinder NLRP3 inflammasome activation by disrupting NF-κB signaling. These mechanisms facilitate parasite propagation [8]. Consequently, inflammasomes play a limited role in host defense against *T. gondii*. In the following sections, we summarize recent advancements in understanding the role of inflammasomes during *T. gondii* infection (Fig. 1).

The NLRP1, NLRP3 and AIM2 inflammasomes are actively involved in the immune response against *T. gondii* infection. The activation of the NLRP1 and the NLRP3 inflammasome contributes significantly to host defense by controlling *Toxoplasma* replication and limiting parasite proliferation. These inflammasomes are critical for generating IL-1β and IL-18, which are essential cytokines that play a key role in combating *Toxoplasma* in both in vitro and in vivo experimental models [12-14]. Studies using knockout mice lacking NLRP3, caspase-1/11, the IL-1 receptor or ASC have demonstrated reduced IL-18 levels, increased parasite growth, and increased mortality rates [13]. IL-18 cytokine production is pivotal in restraining parasite proliferation and enhancing survival in mice deficient in IL-18 or its receptor [13]. Notably, *T. gondii* infection induces both the activation and inhibition of caspases, influencing various outcomes in the responses of murine and human cells to the parasite [15].

### NLRP1 inflammasome

The Lewis rat strain exhibits unexpected resistance to *Toxoplasma* infection, indicating that establishing effective infection through oral or intraperitoneal routes is difficult. This resistance trait is dominantly inherited and linked to bone marrow-derived cells (BMDMs) [14, 16]. Investigations have pinpointed the Toxo1 locus on rat chromosome 10, which spans an 891-kb region that includes the NLRP1 gene and governs Lewis rats' resistance to *T. gondii* [16]. Variations in the NLRP1 gene among rat strains determine differing susceptibilities to *T. gondii* [14, 16]. Silencing NLRP1 in Lewis rat macrophages increases parasite proliferation and provides protection against cell death, whereas overexpression of the NLRP1 variant in BMDMs from susceptible Fischer CDF rats sensitized these cells [14]. Additionally, the NLRP1b allele has been found to enhance the inflammasome response in B6 BMDMs to *Toxoplasma*, independent of the lethal factor proteolysis site [12]. The NLRP1a/Caspase-1 pathway is crucial for mediating parasite-induced macrophage death [16]. Consequently, *Toxoplasma* infection induces NLRP1 inflammasome-dependent release of the proinflammatory cytokine IL-1β in Lewis rat macrophages, inhibiting parasite proliferation [12, 14, 16]. This process triggers typical cellular pyroptosis, facilitating the clearance of intracellular parasites and protecting rats from toxoplasmosis [12, 14].

The dense granule proteins *Tg*GRA35, *Tg*GRA42 and *Tg*GRA43, which are localized in the *T. gondii* parasitophorous vacuole, play critical roles in activating the NLRP1 inflammasome and promoting IL-1β secretion, resulting in pyroptosis of Lewis rat BMDMs and inhibition of parasite proliferation. Infection of macrophages with *Tg*GRA35, *Tg*GRA42 or *Tg*GRA43 knockout parasites significantly reduces cell death rates and increases parasite replication. Additionally, *Tg*GRA42 and *Tg*GRA43 facilitate the proper localization of other *Tg*GRAs, including *Tg*GRA35, to the parasitophorous vacuole membrane [11]. *Tg*GRA35 interacts with the host E3 ubiquitin ligase ITCH, leading to NLRP1 inflammasome activation. Inhibition of proteasome activity or knockout of ITCH prevents *Toxoplasma*-induced pyroptosis in Lewis rat macrophages. Interestingly, the *Tg*GRA35-ITCH interaction contributes to restricting *Toxoplasma* growth in human fibroblasts stimulated with interferon gamma (IFN-γ) [17].

The NLRP1 inflammasome enhances the expression of proinflammatory cytokines such as IL-1β, IL-18 and IL-12. Silencing NLRP1 accelerates host cell death and leads to cell disintegration, which slows the progression of *T. gondii* infection in human monocytic cells [18]. Mice deficient in NLRP1 exhibit increased parasite burdens and higher mortality rates during *T. gondii* infection [13], highlighting the pivotal role of the NLRP1 inflammasome in modulating the pathogenesis of *T. gondii* infection.

### NLRP3 inflammasome

The NLRP3 inflammasome plays a crucial role in clearing *T. gondii* and protecting the host. The activation of the NLRP3 inflammasome in response to *Toxoplasma* infection varies among different host cell types. In murine BMDMs, *Toxoplasma* induces NLRP3 inflammasome activation, leading to the rapid production and secretion of IL-1β [13]. However, primary human macrophages infected with *T. gondii* do not produce IL-1β, possibly because of the downregulation of NLRP3 during monocyte-to-macrophage differentiation and the lack of induction during infection [19]. *Toxoplasma* triggers the Syk-CARD9/MALT-1-NF-κB signaling pathway and activates the NLRP3-caspase-1 inflammasome in primary human monocytes, resulting in IL-1β release in a manner independent of cell death and GSDMD [9]. Additionally, *T. gondii*-induced NLRP3 inflammasome activation in human small intestinal epithelial cells is strongly correlated with p38 MAPK phosphorylation [10].

The activation of the purinergic receptor P2X7 is essential for regulating immune responses against *T. gondii*, reducing parasite load and promoting parasite clearance through canonical NLRP3 inflammasome activation in infected cells [20–23]. In human macrophages infected with *T. gondii*, P2X7 receptor activation decreases the parasite burden by inducing apoptosis in host cells [23]. Deficiency in the P2X7 receptor compromises the ability of both human and mouse macrophages to eliminate *T. gondii*. P2X7 receptor-mediated parasite death occurs alongside host cell apoptosis and operates independently of NO production [20]. ATP-induced activation of P2X7 inhibits *T. gondii* growth in macrophages by promoting NADPH oxidase-dependent ROS production and activating the NLRP3 inflammasome, which triggers IL-1β production through caspase-1 activity and mitochondrial ROS generation [21]. The P2X7 receptor plays a critical role in controlling the parasite load and mitigating tissue damage during virulent *T. gondii* infection [22]. However, in human small intestinal epithelial cells infected with *T. gondii*, P2X7 receptor-mediated NLRP3-dependent IL-1β secretion is crucial for regulating parasite replication. Knockdown of NLRP3, cleaved caspase-1, ASC or the P2X7 receptor significantly decreases *T. gondii*-induced IL-1β production and markedly increases parasite replication [24]. The P2X7 receptor regulates the upregulation of NLRP3 and IL-1β and the downregulation of nitric oxide (NO) following ileitis in *T. gondii*-infected mice. Compared with wild-type mice, the absence of the P2X7 receptor during *T. gondii* infection results in imbalanced ileal inflammatory responses, leading to increased intestinal parasite burdens, extensive inflammation and increased intestinal contractility. Also, compared with serum levels of IL-6, IFN-γ, tissue caspase-1 and NLRP3 in wild-type mice, *T. gondii* infection in P2X7-deficient mice elevates the serum levels of IL-6, IFN-γ and tissue caspase-1, but not NLRP3, [25]. Therefore, the P2X7 receptor, an activator of the NLRP3 inflammasome, plays a crucial role in determining susceptibility to congenital toxoplasmosis and ileitis [25–27].

In addition to ROS and the P2X7 receptor, potassium efflux represents a critical step for NLRP3 inflammasome activation in human monocytes during *T. gondii* infection [19]. The lethal type II strains of *T. gondii*, PRU tachyzoites, RNA and soluble tachyzoite antigen predominantly induce NLRP3 inflammasome activation [28]. Moreover, several immunomodulatory molecules produced by *T. gondii* regulate the activation of the NLRP3 inflammasome to control parasitic growth (Table 1).

**Table 1:** Summary of the role of *Toxoplasma gondii* molecules in modulating NLRP3 inflammasome activation during parasitic infection

Molecule	Description	Cells/animals	Function
Profilin [29]	Induces NLRP3 activation and IL-1β secretion	THP-1 cells	-
*Tg*ROP7 [30]	Interacts with the NACHT domain of NLRP3, leading to an increase in NF-κB and continuous secretion of IL-1β through the IL-1β/NF-κB/ NLRP3 feedback loop	Differentiated THP-1 cells	-
*Tg*GRA9 [31]	Inhibits the formation of the NLRP3 inflammasome by preventing the binding of ASC to NLRP3 in mitochondria, and regulates NLRP3 inflammasome activation	-	Enhances its anti-inflammatory and bactericidal effects through M2 polarization and exhibits anti-septic properties
*Tg*GRA15 [32,33,34]	Induces NLRP3 inflammasome activation or activates NF-κB pathway, resulting in IL-1β secretion	Monocytes	Induces proinflammatory responses, stimulates the host innate immune response
*Tg*GRA7 [35,36]	Activates the MyD88-dependent NF-κB pathway, phosphorylate -ion of *Tg*GRA7-I regulates its interaction with the PYD domain of ASC, facilitating ASC oligomerization and inflammasome activation	-	Induces proinflammatory responses
*Tg*MIF [37]	Triggers NLRP3 inflammasome activation	Hepatocytes	Induces the intense inflammation and pyroptosis, causes fatal liver damage
*Tg*MAPKs [38]	Activate NLRP1 and NLRP3 inflammasome, upregulate expression of IL-18, NLRP1/3, ASC, caspase-1, and downregulate expression of IL-10 and IFN-β	Mice	Increase acute virulence
*Tg*Prx [39]	Promotes alternatively activated macrophages and reduces caspase-1 activity and IL-1β secretion	Macrophages	Modulates immunopathology and promotes parasite replication and survival

*ASC* Apoptosis-associated speck-like protein containing a CARD,* IFN* interferon,* IL* interleukin,* NACHT* nucleotide-binding and oligomerization domain,* NF-κB* nuclear factor kappa-light-chain-enhancer of activated B cells,* NLRP* NLR family pyrin-containing protein,* PYD *pyrin domain, *TgGRA* *T. gondii* dense granule protein, *TgMAPK T. gondii* mitogen-activated protein kinase, *TgMIF* *T. gondii* macrophage migration inhibitory factor, *TgPrx* *T. gondii* peroxiredoxin,* TgROP7* *T. gondii* Rhoptry protein 7

*Toxoplasma gondii* profilin induces NLRP3 activation and IL-1β secretion in THP-1 cells [29]. In that study, at 5 h, a decrease in the messenger RNA (mRNA) expression level of the NLRP3 gene was observed, whereas the protein expression of NLRP3 significantly increased after profilin treatment. The release of IL-1β was found to be significantly reduced by treatment with an NLRP3-specific inhibitor [29]. Rhoptry protein 7 of *T. gondii* (*Tg*ROP7) interacts with the NACHT domain of NLRP3, leading to an increase in NF-κB and continuous secretion of IL-1β through the IL-1β/NF-κB/NLRP3 feedback loop in differentiated THP-1 cells. In that study, deficiency of *Tg*ROP7 in tachyzoites did not influence parasite replication in host cells but attenuated parasite-induced inflammatory activity [30]. *Toxoplasma gondii* dense granule protein (*Tg*GRA9) regulates NLRP3 inflammasome activation to treat host sepsis and interacts with NLRP3, inhibiting the formation of the NLRP3 inflammasome by preventing the binding of ASC to NLRP3 in mitochondria. The C-terminus of *Tg*GRA9 is critical for this regulation due to its association with NLRP3, enhancing its anti-inflammatory and bactericidal effects through M2 polarization and exhibiting anti-septic properties [31]. *Tg*GRA15 has been shown to activate the host innate immune response by mediating monocyte activation of the NLRP3 inflammasome and the production of IL-1β [32, 40]. IL-1β in combination with IFN-γ stimulates inducible NOS (iNOS) expression and NO production in hepatocytes, which markedly reduces the production of indole 2,3-dioxygenase 1, a critical IFN-γ-inducible anti-*Toxoplasma* protein, thereby supporting parasite growth [32]. *Tg*GRA7 and *Tg*GRA15 interact with tumor necrosis factor receptor-associated factors (TRAFs) and activate the NF-κB pathway, strongly inducing NF-κB-mediated proinflammatory responses [33, 35] . Further investigation revealed that *Tg*GRA15-dependent and *Tg*GRA7-dependent NF-κB activation are Myd88-independent and MyD88-dependent, respectively [34, 35]. Protein kinase C alpha (PKCα)-mediated phosphorylation of *Tg*GRA7-I (Ser52) regulates its interaction with the PYD domain of ASC, facilitating ASC oligomerization and inflammasome activation [36]. *Toxoplasma gondii* macrophage migration inhibitory factor (*Tg*MIF) triggers NLRP3 inflammasome activation in hepatocytes, resulting in pyroptosis. The intense inflammation induced by *Tg*MIF restricts the proliferation of intracellular parasites but causes fatal liver damage. In contrast, *Tg*MIF-deficient parasites significantly attenuate liver injury and prolong mouse survival [37]. The *T. gondii* mitogen-activated protein kinases *Tg*MAPK1 and *Tg*MAPK2 are associated with NLRP1 and NLRP3 inflammasome activation in mice infected with the virulent *T. gondii* strain, and parasites lacking *Tg*MAPK1 and *Tg*MAPK2 show reduced acute virulence in mice, characterized by low levels of IL-18, NLRP1/3, ASC and caspase-1 and high levels of IL-10 and interferon beta (IFN-β) transcripts [38]. Treatment with the *T. gondii* redox enzyme peroxiredoxin (*Tg*Prx) induces alternatively activated murine macrophages and reduces caspase-1 activity and IL-1β secretion in macrophages, and in vitro proliferation of the *T. gondii* strain RH increases after pretreatment of macrophages with *Tg*Prx [39].

In addition, the ginsenoside Rh2 attenuates neuropathological damage and neuronal apoptosis in the cortical tissue of mice during *T. gondii* infection. Studies have shown that the ginsenoside Rh2 binds to *T. gondii* calcium-dependent protein kinase 1 and NLRP3 and decreases the expression of the specific microglial markers ionized calcium-binding adapter molecule 1 and NLRP3 inflammasome-related proteins, leading to the inhibition of the NLRP3 inflammasome and a protective effect against *T. gondii* infection-induced neuronal injury in microglia [41].

### Other inflammasomes

PRU genomic DNA (gDNA) specifically activates the AIM2 inflammasome in peritoneal macrophages, leading to increased susceptibility to *T. gondii* PRU infection in mice lacking AIM2 [28]. Activation of the AIM2 inflammasome results in the release of caspase-1, mature IL-1β and GSDMD cleavage, contributing to the pyroptosis of placental cells induced by *T. gondii* [42].

The activation of the immune response and inflammation in toxoplasmosis is associated with NLRP12. In one study, the transcription of the NLRP12, caspase-3, caspase-1, IL-1β, IL-18 and ASC genes was upregulated in nucleated cells within the peritoneal fluid, and a significantly greater level of the NLRP12 protein was observed in the *T. gondii*-infected BALB/c murine model than in the control group [43].

The role of inflammasome-related genes in the innate immune response to *T. gondii* infection is crucial. *Toxoplasma gondii* infection led to time-dependent increases in the mRNA expression of inflammasome sensors such as NLRP1, NLRP3, NLRC4, NLRP6, NLRP8, NLRP13, AIM2, and NAIP, along with elevated mRNA levels of the adaptor proteins ASC, caspase-1, and IL-1β in THP-1 macrophages [44]. Additionally, soluble antigens from *T. gondii* strain RH tachyzoites time-dependently upregulated NLRP1, NLRP3, NLRC4, and AIM2 gene expression; downregulated IL-1β and IL-18; and increased IL-1β release in THP-1 macrophages [45]. In FHs Int 74 cells infected with *T. gondii*, the mRNA expression of NOD2, NLRP3, NLRP6, and NAIP1 significantly increased, whereas the expression of NLRP2, NLRP7, and CIITA decreased. *Toxoplasma gondii* infection activated the NLRP3, NLRP6, and NLRC4 inflammasomes in these cells, resulting in the production of IL-1β, IL-18, and IL-33. Interestingly, NLRP6 inflammasome activation in response to *T. gondii* was independent of the MAPK pathway in FHs 74 Int cells [10]. Furthermore, *T. gondii* infection of human placental cells induced the activation of the NLRP1, NLRP3, NLRC4, and AIM2 inflammasomes and increased ASC expression and the levels of mature IL-1β, cleaved caspase-1, and GSDMD, leading to pyroptosis in placental trophoblasts and amniotic cells. The administration of ROS scavengers, a CatB inhibitor, or inflammasome-specific siRNA reversed adverse pregnancy outcomes in a *T. gondii*-infected pregnant murine model [42]. Moreover, Caspase-8 enhances NF-κB family member IκB kinase phosphorylation, facilitates c-Rel nuclear translocation, and regulates IL-12 and IL-1β expression. Administration of exogenous IL-12 restores monocyte recruitment and effectively controls parasite burden in Ripk3^–/–^Casp8^–/–^ mice [46]. Further investigation revealed that caspase-8 regulates IL-1β secretion via a mechanism independent of gasdermin in *T. gondii*-infected human monocytes. Simultaneous pharmacological inhibition of caspase-8 and RIPK1 in primary monocytes reduces IL-1β release without affecting cell viability or parasite infection [47].

### Noncanonical inflammasomes

Protective immunity against *T. gondii* invasion is generated through inflammasome signaling, which suppresses the production of type I IFN (IFN-I). This suppression is crucial for IFN-γ production by natural killer cells and the recruitment of inflammatory monocytes to the infection site [48]. Mechanistically, inflammasome-coupled IL-1β signaling via caspase-1/caspase-11 triggers the expression of the negative regulator SOCS1. SOCS1 binds to INF regulatory factor 3, inhibiting IFN-I production [28]. Additionally, *T. gondii*-induced activation of the caspase-1/caspase-11-dependent inflammasome and IL-18 is essential for sustaining robust CD4^+^ T_H_1 IFN-γ responses in a mouse model of *T. gondii* infection in the absence of Toll-like receptor 11 (TLR11) [49].

Caspase-11 plays a protective role by increasing inflammation early in infection to mitigate disease severity later in toxoplasmosis. Mice lacking caspase-11 exhibit notably lower cytokine production, reduced pathogenesis and decreased inflammation during the acute phase of *T. gondii* strain ME49 infection. Conversely, caspase-11 deficiency results in increased neuroinflammation and increased accumulation of tissue cysts in the brain during the chronic phase of infection [50].

In conclusion, the activation of inflammasomes by *T. gondii* varies depending on the host. In addition to the NLRP1 and the NLRP3 inflammasome, AIM2, NLRC4, NLRP6, NLRP8, NLRP12, NLRP13 and NAIP inflammasomes, as well as noncanonical inflammasomes, have been investigated both in vivo and in vitro during *T. gondii* infection. *Toxoplasma gondii* manipulates inflammasome activation and inhibition, influencing immune responses and inflammation. These inflammasomes are crucial for regulating the *T. gondii* burden, controlling parasite growth and proliferation and managing parasite-induced cell death. Therefore, inflammasomes play a vital role in orchestrating innate immune responses against *T. gondii* infection.

## *Plasmodium*

Malaria is a lethal infectious disease caused by protozoan parasites of the genus *Plasmodium* that affects millions of people globally. In 2022, approximately 249 million cases and 608,000 associated deaths were reported in 85 countries [51]. There are four primary human-infecting *Plasmodium* species, namely *Plasmodium ovale*, *P. malariae*, *P. vivax*, and *P. falciparum*, of which the most significant are *P. falciparum* and *P. vivax*. The infection process begins when hepatocytes are invaded by sporozoites, followed by asexual reproduction within liver cells. *Plasmodium* must develop and replicate within hepatocytes before progressing to the life-threatening blood stage of the disease. Merozoites, which are released from hepatocytes, periodically infect erythrocytes, leading to malaria-associated pathologies**.** Typical symptoms include intermittent chills, fever and sweating, potentially resulting in spleen enlargement; anemia; and, in severe cases, damage to vital organs such as the brain, liver and kidneys or even multisystem failure of the circulatory and respiratory systems, ultimately leading to death. Placental malaria is associated with significant inflammation, leading to complications such as abortion, preterm delivery and intrauterine growth restriction. Cerebral malaria is a serious neurological complication caused by *P. falciparum* infection and is characterized by parasite sequestration, inflammatory cytokine production and vascular leakage. Murine experimental cerebral malaria (ECM) strains have been generated using *Plasmodium berghei* ANKA (*Pb*A) strain and *Plasmodium chabaudi*, as malaria parasites infecting humans do not infect mice.

It has been established that innate immune responses in the liver can modulate *Plasmodium* infection, potentially reducing the incidence and severity of clinical malaria [52–56]. The robust IFN-I response enhances innate immune control, resulting in delayed progression to the blood stage of malaria, reduced parasitemia and increased survival in mice [53, 57]. Thus, inducing type I IFNs to limit *Plasmodium* infection in the liver decreases the extent and impact of clinical malaria [57].

**Fig. 2:**
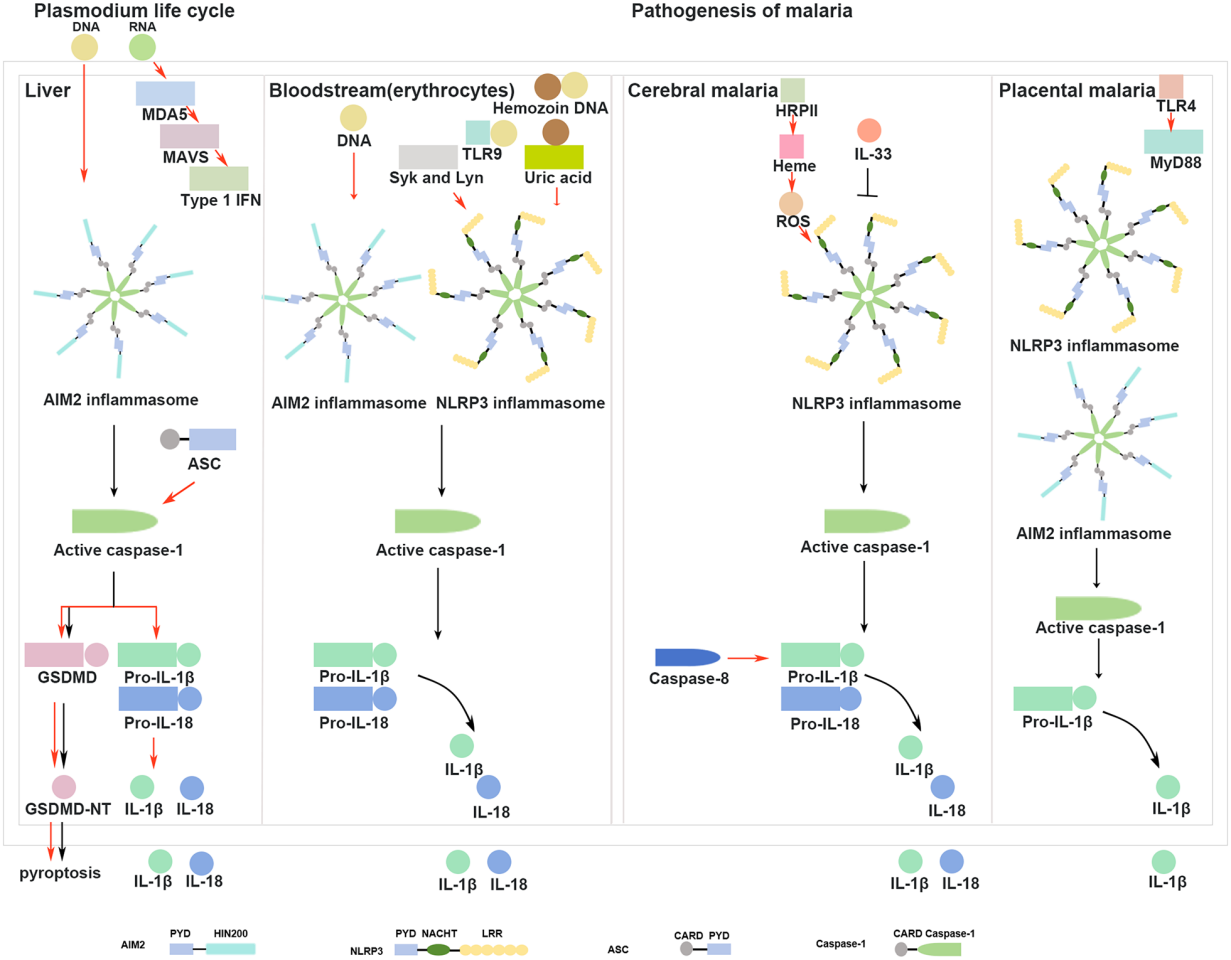
Role of inflammasomes in modulating the immune response against *Plasmodium* infection. The AIM2 and the NLRP3 inflammasome are predominantly activated during the *Plasmodium* life cycle and the pathogenesis of malaria. In liver cells infected by *Plasmodium*, *Plasmodium* DNA is detected by AIM2 receptors in hepatocytes, and ASC facilitates complete caspase-1 processing, enhances GSDMD-mediated pyroptotic cell death and enables the secretion of mature IL-1β and IL-18, thereby improving the overall control of *Plasmodium* infection in the murine liver [85]. In erythrocytes infected by *Plasmodium*, when complexed with the malarial pigment hemozoin, *Plasmodium* DNA induces TLR9 translocation, post-phagocytosis and hemozoin (Hz) and DNA dissociation [64]. DNA and Hz induce AIM2 and NLRP3 inflammasome activation, respectively [64]. Hz activates the NLRP3 inflammasome through Lyn and Syk kinases [68]. Uric acid may enhance the initial host inflammatory response to hemozoin via NLRP3 inflammasome activation [71]. Caspase 8 and the NLRP3 inflammasome regulate host inflammatory responses in cerebral malaria. AIM2 and the NLRP3 inflammasome contribute to the pathology associated with placental malaria [65]. ASC, Apoptosis-associated speck-like protein containing a CARD; GSDMD, N-terminal of gasdermin D; IFN, interferon; IL, interleukin; MDA5, melanoma differentiation-associated protein 5; MAVS, mitochondrial antiviral signaling protein; NLRP, NLR family pyrin-containing protein; TLR, Toll-like receptor

The rupture of infected erythrocytes triggers an inflammatory response induced by parasitic factors such as *Plasmodium* PAMPs or DAMPs, which leads to the production of inflammatory cytokines such as TNF alpha (TNF-α), IFN-γ, IL-1β and IL-18. *Plasmodium* components, including gDNA, RNA, hemozoin (Hz) and glycosylphosphatidylinositol, are captured by immune cells, stimulating the activation of inflammasomes such as AIM2, NLRP3 or NLRC4 and resulting in cytokine release, which manifests as cyclic paroxysm and high fever. Recognition of *Plasmodium* glycosylphosphatidylinositol by host TLR2 and TLR4, cytosolic RNA by TLR7 and parasite DNA and Hz-DNA complexes by TLR9 has been noted [58–61]. NLRs and AIM2 significantly contribute to inflammasome activation [56]. During *Plasmodium yoelii* infection, *Plasmodium* gDNA, RNA and Hz lead to the activation of the AIM2 and the NLRP3 inflammasome in dendritic cells and macrophages [62]. High concentrations of immunocomplexes containing parasite DNA induce the AIM2 and NLRP3 inflammasome assembly, caspase-1 activation and IL-1β secretion in monocytes from infected patients, thereby stimulating systemic inflammation during acute malaria episodes [63]. Systemic inflammation resulting from the parasite often dictates the clinical presentation. Recent studies on the role of host inflammasomes in preventing *Plasmodium* parasite infection are summarized in the following sections (Fig. 2).

### AIM2 inflammasome

The AIM2 inflammasome and its components are involved in the inflammatory responses of *Plasmodium*-infected hepatocytes, erythrocytes, liver and placenta. In macrophages**,** plasmodial gDNA on the surface of Hz activates the AIM2 inflammasome and induces mature IL-1β production [64], as already demonstrated in studies of *T. gondii* infection [28, 42]. In *Plasmodium*-infected hepatocytes, *Plasmodium* DNA is detected by cytosolic AIM2 receptors, leading to the activation of inflammasome-mediated caspase-1, GSDMD cleavage and pyroptotic cell death, contributes to the elimination of *Plasmodium*-infected hepatocytes and the control of malaria infection in the liver [52]. *Plasmodium*-infected erythrocytes induce AIM2 inflammasome activation and IL-1β production [64]. Furthermore, the activation of the AIM2 inflammasome and caspase-1-dependent signaling negatively regulate host immune responses against lethal *P. yoelii* YM infection, and parasitemia is reduced in infected AIM2- and caspase-1-deficient mice compared with wild-type mice. Mice lacking AIM2 and caspase-1 are completely resistant to *P. yoelii* YM infection [62]. The AIM2 inflammasome also affects fetal growth and viability and placental IL-1β production during murine placental malaria and contributes substantially to placental malaria-related pathogenesis [65].

### NLRP3 inflammasome

The role of the NLRP3 inflammasome is important in terms of the ability of the host to resist *Plasmodium* infection at three key parasitic sites: erythrocytes, the brain and the placenta.

#### Erythrocytes

In *Plasmodium*-infected erythrocytes, the intraerythrocytic parasite *Plasmodium* produces Hz, a crystalline detoxification product of hemoglobin, which is released into the circulation during erythrocyte lysis and facilitates further infection of naive red blood cells. The potent proinflammatory effect of Hz through inflammasome activation is implicated in *Plasmodium*-associated pathologies [66]. Previous studies demonstrated that the malarial pigment Hz transports plasmodial DNA to a subcellular compartment accessible to TLR9, inducing inflammatory signals [64]. The stimulation of TLR9 by natural Hz depends on the presence of *Plasmodium* DNA on the crystal surface rather on than the Hz itself [67], suggesting a collaborative role of Hz and DNA in inducing systemic inflammation during malaria infection. Hz is inherently immunoreactive, activating the NLRP3 inflammasome and leading to the secretion of IL-1β and IL-18 [57, 64, 66–69]. The inhibition of phagocytosis, potassium efflux and NADPH oxidase activity in THP-1 cells and BMDMs blocks Hz activity [66]. Research has shown that the activation of the NLRP3 inflammasome is induced by Hz via the Src kinase Lyn and the spleen tyrosine kinase Syk in vivo and in vitro [68]. Genetic, chimeric and pharmacological methods have demonstrated that the parasite-derived crystal Hz depletes conventional CD8α^+^ type 1 dendritic cells and also impairs their function in an NLRP3 inflammasome-dependent manner in vivo, resulting in decreased CD4^+^ T follicular helper cell differentiation and weakened anti-*Plasmodium* humoral immunity [70]. Based on these study results, NLRP3 inflammasome-mediated immune recognition and the inflammatory response are considered to be elicited by Hz [66, 71, 72].

Activation signals in addition to Hz, such as uric acid, extracellular ROS production, potassium efflux, and Syk and NADPH oxidase-2 activation, induce NLRP3 inflammasome activation [71, 73, 74]. First, the levels of uric acid, the degradation products of the purine derivatives hypoxanthine and xanthine that accumulate in *Plasmodium*-infected erythrocytes, are increased in the bloodstream following the rupture of infected red blood cells during severe malaria in humans and mice [75–77]. Uric acid may enhance the initial host inflammatory response to Hz via NLRP3 inflammasome activation, which is characterized by neutrophil recruitment and inflammatory cytokine and chemokine production in vivo. Nevertheless, inflammasome activation by synthetic Hz is mitigated by allopurinol, an inhibitor of uric acid synthesis [71]. In contrast, uric acid does not play a role in NLRP3 activation by synthetic Hz β-hematin in THP-1 cells [66]. Second, heme, a marker of hemolysis or extensive cell damage, activates the NLRP3 inflammasome and promotes IL-1β synthesis in macrophages by inducing Syk and NADPH oxidase-2 activation, mitochondrial ROS production, and potassium efflux, which play critical roles in malaria pathogenesis [73]. Finally, increased plasma xanthine oxidase activity is associated with elevated levels of inflammatory cytokines and the onset of cerebral malaria in malaria patients [74]. Subsequent investigations demonstrated that extracellular ROS generated by xanthine oxidase activate the NLRP3 inflammasome and promote the processing of pro-IL-1β, triggering a potent inflammatory response in primary human monocyte-derived macrophages [74].

#### Brain

In rodent cerebral malaria, the role of the NLRP3 inflammasome is contentious. One study revealed that mice lacking NLRP3 presented delayed onset of cerebral malaria, whereas those deficient in caspase-1, the adaptor protein ASC, or the IL-1 receptor presented symptoms similar to those of wild-type mice. Thus, in *Pb*A-induced murine cerebral malaria, NLRP3 functions independently of the inflammasome and the IL-1 receptor [78]. Another study revealed that infection of NLRP3-deficient mice with *Pb*A sporozoites suppressed cerebral malaria pathogenesis without affecting parasitemia [66]. Similarly, the absence of NLRP3 or IL-1β resulted in increased survival and lower body temperature in mice during *Plasmodium chabaudi adami* DS infection [68], demonstrating partial protection to the ECM. Moreover, the activation of the inflammasome-associated components IL-1β and IL-18 through caspase-1 does not influence *Pb*A-induced immunopathology [79]. These studies demonstrate that NLRP3, ASC, caspase-1, IL-18 or the IL-1 receptor have only minor or no effects on the control of parasitemia and the development of the mouse ECM [68, 80], which is contrary to findings in *T. gondii* [10]. However, mice deficient in NLRP3 and caspase-1 are resistant to *P. yoelii* YM infection and present lower levels of parasitemia than wild-type mice do, indicating that NLRP3 and caspase-1 inflammasome activation negatively modulates host immune responses to resist lethal *P. yoelii* YM infection [62].

In one study, the expression of IL-33 at both the gene and protein levels decreased in the brain during fatal ECM [81]. Treatment with IL-33 following *Pb*A-induced murine cerebral malaria reduced NLRP3 inflammasome formation and IL-1β secretion in microglia and intracerebral monocytes during the acute recovery phase. These findings highlight the role of the IL-33-NLRP3 pathway in neuroinflammation and cerebral pathology, which impacts the efficacy of antimalarial drug treatment for cerebral malaria [81].

In brain endothelial cells, NLRP3 mRNA expression is upregulated after exposure to *Pb*A [78]. The virulence of histidine-rich protein II, a diagnostic and prognostic marker for *P. falciparum* infection, lies in its delivery of numerous hemes to cerebral microvascular endothelial cells, causing iron toxicity, ROS production and activation of the NLRP3 inflammasome, leading to IL-1β production. This sequence disrupts human cerebral microvascular endothelial barrier integrity, increases endothelial permeability and promotes vascular inflammation and edema [79].

#### Placenta

The NLRP3 inflammasome impairs fetal growth in pregnant mice with placental malaria and may contribute significantly to the development of placental malaria-related pathology. Anakinra, a pharmacological IL-1 receptor antagonist, has shown promise in improving pregnancy outcomes by promoting fetal growth and reducing resorption in experimental placental malaria [65].

### Other inflammasomes

Investigations on the impact of single nucleotide polymorphisms on hematological and clinical parameters through multivariate analysis have revealed that the NLRP1 inflammasome receptor is crucial in determining the clinical outcomes of *P. vivax* malaria, including fever, anemia and thrombocytopenia. IL-1β rs1143634 is notably associated with patient parasitemia, whereas IL-18 rs5744256 appears to play a protective role against anemia development.

The NLRP12 inflammasome is present in monocytes from patients with febrile *P. vivax* infection, and activation of the NLRP12 inflammasome by *Plasmodium* is likely a pivotal event in promoting systemic IL-1β production and heightened sensitivity to bacterial superinfection during malaria sepsis [82].

Caspase-11, caspase-4 and caspase-8 are activated in both *Pb*A- and *P. chabaudi*-infected mice and in patients infected with *P. vivax* and *P. falciparum*. Both caspase-1 and caspase-8 are involved in the production and secretion of IL-1β and TNF-α, thereby contributing to increased susceptibility to septic shock and the development of ECM in *P. chabaudi*-infected mice and *P. berghei*-induced ECM [83]. In malaria patients, caspase-1, caspase-4, caspase-8 and GSDMD are activated in monocytes [74, 82, 83], which may contribute to the overall increased levels of IL-1β and immunopathogenesis during *P. vivax* and *P. falciparum* infections [74, 82, 83]. The IL-18 system is integral to the pathogenesis of *P. falciparum*, with both IL-18- and IL-18-binding proteins correlating positively with disease severity, parasitemia and endothelial cell activation in the plasma of malaria patients [84].

In the liver, inflammasome-mediated caspase-1 activation further enhances the control of *Plasmodium*, thus reducing the frequency and severity of clinical malaria. However, caspase-1 processing in both mouse and human hepatocytes is incomplete, preventing the secretion of mature IL-1β or IL-18. This incomplete processing is attributed to the inherently low expression of the inflammasome adaptor molecule ASC in murine hepatocytes. In contrast, transgenic overexpression of ASC in hepatocytes facilitates complete caspase-1 processing, enhances GSDMD-mediated pyroptotic cell death and enables the production of proinflammatory cytokines, such as mature IL-1β and IL-18, which are otherwise absent, thereby improving the overall control of *Plasmodium* infection in the murine liver [85].

Caspase-12 attenuated parasite clearance in blood-stage malaria and impacted susceptibility to cerebral malaria by limiting immune control of parasite replication and reducing cerebral hyperinflammation in a cerebral malaria model. Caspase-12 competes with a key regulator of NF-κB for binding to IκB kinase-α/β, blocking the formation of the IκB kinase complex and inhibiting downstream NF-κB-dependent transcriptional activation, separate from the caspase-1 inflammasome [86].

Recent evidence suggests that different host inflammasomes are involved in distinct stages of *Plasmodium* infection. The AIM2 inflammasome regulates *Plasmodium* control in the liver. In erythrocytes infected by *Plasmodium*, the NLRP3, NLRP12 and AIM2 inflammasomes participate in immune recognition and inflammatory responses. The NLRP3 inflammasome, caspase-4, caspase-8, caspase-11 and caspase-12 are crucial for regulating host inflammatory responses in cerebral malaria. The AIM2 and the NLRP3 inflammasome contribute to the pathology associated with placental malaria [65]. Inflammasomes play key roles in immune recognition, inflammation, parasite control, the development of *Plasmodium*-related pathologies and the severity of clinical malaria.

## Comparison of the role of inflammasomes in the host response to apicomplexan parasites

The intracellular protozoan parasites *Toxoplasma* and *Plasmodium* cause systemic infections in a wide range of cell and tissue types. Multiple inflammasomes, including the AIM2, NLRP1, NLRP3 and NLRP12 inflammasomes and caspase-8, which play critical roles in initiating the inflammatory response to resist *Toxoplasma* and *Plasmodium* infections, have been studied. Research on the role of inflammasomes against *Toxoplasma* and *Plasmodium* infections has been conducted not only at the cellular level but also in animal models. The NLRP1 inflammasome pathway triggers cellular pyroptosis and directs resistance to toxoplasmosis in Lewis rats, whereas the NLRP1 receptor is crucial in determining the clinical outcomes of *P. vivax* malaria [87]. Moreover, the NLRP3 inflammasome mediates immune recognition and the inflammatory response and eliminates two pathogens to protect the host, modulating the pathogenesis of *Toxoplasma* and *Plasmodium* infections. The role of the NLRP3 inflammasome in rodent cerebral malaria is controversial. The activation signals, including ROS, ATP, P2X7 receptor, potassium efflux and *T. gondii* molecules, induce NLRP3 inflammasome activation in response to *Toxoplasma* infection; however, Hz, uric acid and extracellular ROS production; potassium efflux; and Syk and NADPH oxidase-2 activation induce NLRP3 inflammasome activation in the context of *Plasmodium* infection [64, 71, 73, 74]. Whether the activation of the P2X7 receptor can activate the NLRP3 inflammasome against *Plasmodium* infection remains unknown. Furthermore, the AIM2 inflammasome, which is activated by the gDNA of two pathogens, contributes to pyroptosis in *T. gondii*-infected placental cells and is involved in the inflammatory responses of *Plasmodium*-infected hepatocytes, erythrocytes, the liver and the placenta. Caspase-1 and caspase-8 are involved in the production and secretion of IL-1β during *Toxoplasma* and *Plasmodium* infections.

Research on the host inflammasome of *Toxoplasma* and *Plasmodium* differs. First, in terms of the types of inflammasomes studied, the NLRP1 and the NLRP3 inflammasome have been studied more in the context of *Toxoplasma* infection, and the AIM2 and the NLRP3 inflammasome have been studied against *Plasmodium* infection, with more studies on the NLRP3 inflammasome. Future studies are needed to investigate the role of other inflammasomes. The noncanonical inflammasome caspase-11 plays a protective role in *T. gondii* infection, promoting an inflammatory response early in infection and reducing disease severity later in infection [50], but the role of noncanonical inflammasomes underlying *Plasmodium* infection is still unclear. Second, in terms of parasite effector proteins, several molecules in *Toxoplasma gondii* are involved in NLRP3 inflammasome regulation, GRA9 negatively regulates inflammasome activation, and the rest activate the inflammasome. *Plasmodium* Hz, DNA and RNA induce inflammasome activation. Further studies are needed to understand the specific role of other parasitic products in NLRP3 inflammasome regulation.

In addition to *Plasmodium* and *Toxoplasma*, *Cryptosporidium* also belongs to the phylum Apicomplexa. There is little research on *Cryptosporidium*. Epithelial cell-intrinsic NLRP6/caspase-1-mediated secretion of IL-18 is required for the control of *Cryptosporidium* [88]. Compared with wild-type mice, mice deficient in core components of the inflammasome are more susceptible to *Cryptosporidium parvum* and *C. tyzzeri* infections [88, 89]. Research has shown that the NLRP1, NLRP3 and AIM2 inflammasomes are associated with the control of the related apicomplexan parasites *Toxoplasma* and *Plasmodium* but are not required for the control of *Cryptosporidium*. This discrepancy may reflect the pronounced differences in the tissue and host-cell tropisms of apicomplexan parasites, with *Cryptosporidium* being restricted to enterocytes, whereas *Toxoplasma* and *Plasmodium* cause systemic infections in a wide range of cell types [88].

## Conclusions

Inflammasomes, recognized as critical signals in innate immunity, control parasite burden, manage parasite-induced cell death and play crucial roles in the onset and progression of toxoplasmosis and malaria. *Toxoplasma gondii* has evolved multiple strategies to evade host immune defenses; however, the underlying mechanisms of these strategies are still not well understood. Further research is needed on how *T. gondii* bidirectionally regulates inflammasomes and modulates the magnitude of inflammasome activation and whether other apicomplexan parasites bidirectionally regulate inflammasomes to balance inflammation and promote chronic disease. It is increasingly important to maintain the proinflammatory effects of inflammasomes in a curative rather than pathogenic state during infections. A deeper understanding of how parasitic infections regulate inflammasomes could significantly improve our knowledge of the host immune response to apicomplexan infections. Insights into the regulatory mechanisms of inflammasomes are essential for advancing drug discovery, adjuvant development and vaccine production.

## Data Availability

No datasets were generated or analysed during the current study.

## Abbreviations

ASC: Apoptosis-associated speck-like protein containing a CARD
BMDMs: Bone marrow-derived cells
CARD: Caspase-activating and recruitment domain
DAMPs: Damage-associated molecular patterns
ECM: Experimental cerebral malaria
GSDMD: Gasdermin D
GSDMD-NT: N-terminal of gasdermin D
Hz: Hemozoin
IFN: Interferon
IL: Interleukin
iNOS: inducible NOS
LPS: Lipopolysaccharide
LRR: Leucine-rich repeat
MAVS: Mitochondrial antiviral signaling protein
MDA5: Melanoma differentiation-associated protein 5
mRNA: messenger RNA
NACHT: Nucleotide-binding and oligomerization domain
NF-κB: Nuclear factor kappa-light-chain-enhancer of activated B cells
NLR: Nucleotide binding oligomerization domain-like receptor
NLRP: NLR family pyrin-containing protein
NO: nitric oxide
PAMPs: Pathogen-associated molecular patterns
*Pb*A: *Plasmodium berghei* ANKA strain
PKCα: Protein kinase C alpha
PRRs: Pattern recognition receptors
PYD: Pyrin domain
ROS: Reactive oxygen species
*Tg*GRA: *T. gondii* Dense granule protein
*Tg*MAPK: *T. gondii* Mitogen-activated protein kinase
*Tg*MIF: *T. gondii* Macrophage migration inhibitory factor
*Tg*Prx: *T. gondii* Peroxiredoxin
*Tg*ROP7: *T. gondii* Rhoptry protein 7
TLR11: Toll-like receptor 11
TNF-α: TNF alpha
TRAFs: Tumor necrosis factor receptor-associated factors
